# Incidence of medication errors in a Moroccan medical intensive care unit

**DOI:** 10.1186/1755-7682-4-32

**Published:** 2011-10-04

**Authors:** Naoual Jennane, Naoufel Madani, Rachida OuldErrkhis, Khalid Abidi, Ibtissam Khoudri, Jihane Belayachi, Tarik Dendane, Amine Ali Zeggwagh, Redouane Abouqal

**Affiliations:** 1 Medical Intensive Care Unit, Ibn Sina University Hospital, Rabat, Morocco; 2 Emergency Poison Unit, Hygiene Institute, Rabat, Morocco; 3 Laboratory of Biostatistics, Clinical and Epidemiological Research, Faculty of Medicine, Rabat, Morocco

**Keywords:** Medication Error, Intensive care unit, Adverse drug event, potential adverse drug event

## Abstract

**Background:**

Medication errors (ME) are an important problem in all hospitalized populations, especially in intensive care unit (ICU). The aim of the study was to determine incidence, type and consequences of ME.

**Materials and methods:**

Prospective observational cohort study during six weeks in a Moroccan ICU. Were included all patients admitted for > 24 hours. ME were collected by two reviewers following three methods: voluntary and verbally report by medical and paramedical staff, chart review and studying prescriptions and transcriptions. Seriousness of events was classified from Category A: circumstances or events that have the capacity to cause error, to Category I: patient's death.

**Results:**

63 patients were eligible with a total of 509 patient-days, and 4942 prescription. We found 492 ME, which incidence was 10 per 100 orders and 967 per 1000 patient-days. There were 113 potential Adverse Drug Events (ADEs) [2.28 per 100 orders and 222 per 1000 patient-days] and 8 ADEs [0.16 per 100 orders and 15.7 per 1000 patient-days]. MEs occurred in transcribing stage in 60%cases. Antibiotics were the drug category in 33%. Two ADEs conducted to death.

**Conclusion:**

MEs are common in Moroccan medical ICU. These results suggest future targets of prevention strategies to reduce the rate of ME.

## Background

Iatrogenic injuries occur commonly in the health care system. In recent years, medication error events received considerable attention because of its substantial mortality, morbidity, and additional health care costs. Many reports indicated that nearly one third of adverse drug events (ADEs) are associated with medication errors and are thus preventable [1-6]. The frequency of these events was described over several studies. According to Brennan et al [7] 3.7% of hospitalized patients experienced an adverse event related to medical therapy in 1984. Of these iatrogenic injuries, 69% were preventable. Later, Lazarou et al [8] estimated that over 100.000 hospitalized patients in 1994 had fatal adverse drug reactions. Bates et al [9] reported 5 medication errors per 100 medication orders; only 7 in 100 medication errors had significant potential for harm. Other studies largely confirmed that ADEs are common; and have important economic and human consequences [3,10] as patients suffering from ADEs have about three times the mortality rate compared with matched controls, with an increase of about two days in length of stay, and an increased cost of more than $US2000 of admission. Thus, identification of ADEs and medication errors appears to be a crucial first step in improving patient safety, although the approach seems to be different in research and routine care [11].

Estimates of ADEs rates vary substantially depending on the setting and the data sources used. Intensive care units (ICUs) can be considered as an optimal location for developing voluntary reporting incentives based on the frequency of events. In fact, ICU patients may be at higher risk for ADEs [12] because of the higher exposure to medicines and the weaker health conditions compared with other patients. Cullen et al [13] reported twice combined incidence of preventable and potential ADEs that rate in non ICU areas. Specific improvements in the medication ordering and processing system may reduce the risk of errors. Studies have demonstrated that some of these interventions can be effective such us physician computer order entry and a computerized clinical decision support program [14-20].

In medical community, few epidemiological data are available regarding the incidence, type and causes of medication errors in ICUs. Less information is available concerning medication errors and ADEs in developing countries and in Morocco. In this field, a pilot project built by the World Alliance for Patient Safety in collaboration with the World Health Organization program for International Drug Monitoring was initiated in 2007. The Moroccan Pharmacovigilance Centre was assigned as project coordinator. As part of this project, a prospective study in Moroccan medical ICU was initiated showing 7.7% medication errors for 1000 patient-days [12].

The aim of the present study was to evaluate incidence, type and consequences of medication errors in a Moroccan medical ICU.

## Methods

### Study design and setting

It was a prospective observational study conducted in a 12 bed medical ICU of Rabat University Hospital during six weeks of September 2009. Rabat University Hospital is a major teaching medical centre and the biggest hospital in North Africa. It is referral for habitants in Western-North Morocco.

The 12 bed medical ICU admits approximately 550 adult patients annually in order to treat medical urgent diseases such us sepsis, metabolic and neurological illness. Elsewhere, surgical illness, complications after surgeries, neonates and burn patients are treated in specialized units in Rabat University Hospital.

Before initiating this study, we gained the support of the leader ship of nursing, pharmacy, medical staff, and administration who received informal seminars that emphasized the roles of complex systems and human factors in predisposing to error, as opposed to individual blame. We stressed the importance of understanding the epidemiology and causes of error, and reinforced the multidisciplinary nature of systems improvement. The study was conducted after approving all aims, conditions and methods by the local ethic committee named: the "*comité d'éthique de la recherche biomédicale*" of Rabat faculty of medicine and pharmacy. Informed consent was obtained from all conscious patients and from families of comatose patients.

### Patients

The study included consecutive patients hospitalized in medical ICU during the study period at any degree of disease severity, independently of their age and gender. Patients excluded from the study were those transferred to other units or died in medical ICU before registering any medication error.

The following data were recorded for all patients using the patient's medical chart review: patient characteristics (age and gender), Acute Physiology and Chronic Health Evaluation II (APACHE II) score [21], diagnoses, duration of hospitalization, and outcome. Patients' related data was confidential, and was destroyed after collecting information and constituting data base.

### Inpatient Medication Use Process

At the time of the study, medication orders were handwritten by physicians with guidelines for prescribers. Medication supply to the patients was done by the nursing staff from stock bottles of commonly used medicines in the ward. The medicine stock bottles were held in a central locked area. It was the chief of the nursing stuff who is in charge of the stock ward. In accordance with hospital policy, patients were also encouraged to bring their own current medications into hospital. When a charted medication was not held on the ward or available as the patient's own supply, the item was dispensed from the hospital pharmacy.

For each administration, the date and the time of medication administration were recorded on the administration section of the medication chart.

### Identification of Medication-Related Events

One physician (N.J) trained all data collectors, who were nurses, pharmacist, and physicians, in an identical manner. During training, the unique perspectives of these different disciplines were shared to maximize appreciation of potential error types and to develop a comprehensive, uniform approach to error detection. Data collectors worked 5 days per week, with recording of weekend data on Mondays for patients still hospitalized. Two methods of data collection were combined: observational study in which investigators participated in daily physician rounds and monitored ordering and transcribing medication. Solicited reports from health professionals were the second method of incident identification.

Data collectors identified medication errors, potential ADEs and ADEs, by voluntary and verbally solicited reports from house officers, nurses and pharmacists; and by medication order sheet, medication administration record, and chart review of all hospitalized patients on study wards. On a given day, 1 data collector was assigned to the study ward based on individual availability. Data collected for each incident included name, dose, route and category of drug, point in the system where the error occurred, and type of error.

Reliable detection of medication errors requires cooperation and engagement of the staff, which depends in large measure on reducing suspicion and fear of reporting.

### Review Process

A physician (N.J) and a pharmacist (R.O) independently reviewed suspected ADEs and potential ADEs and classified them as ADEs, potential ADEs, medication errors, and rule violations. The physician reviewers rated ADEs and potential ADEs according to the severity of injury to the patient. The 2 evaluators resolved all disagreements through discussion and consensus.

### Definitions

The following definitions were those from the National Coordinating Council for Medication Error Reporting and Prevention [22]:

A *medication error *(ME) was defined as any error occurring in the medication process (ordering, transcribing, dispensing, administering, and monitoring). An example is an order written for amoxicillin without a route of administration. MEs are the broadest category and while most have little potential for harm, some do and are either potential ADEs or preventable ADEs depending on whether an injury occurred.

*Potential ADEs *was defined as a medication error with the potential to cause any injury but which does not actually cause any injury, either because of specific circumstances, chance or because the error was intercepted and corrected (e.g. error was intercepted before the patient was affected or the patient received a wrong dose but no harm occurred) (Table 1). All potential ADEs are MEs but not all MEs are potential ADEs.

**Table 1:** Adverse drug events (ADEs) and potential ADEs: study definitions and examples [24]

Example	Definitions	Event type
**Medication error (ME)**	Any injury in any stage of the medication process, including ordering, transcribing, dispensing, administering or monitoring	A dose of non-critical medication is not given
**Potential ADE**	An incident with potential for injury; all potential ADEs are ME	An order was written for an overdose of medication but the mistake was intercepted by the pharmacy
**ADE**	Injury due to a drug	Drug rush
Preventable	Due to an error	Coma due to over dose of sedative
Non preventable (adverse drug reaction)	Injury, but no error involved	Allergic reaction in patient not known to be allergic

*ADEs *were defined as any injury resulting from medical interventions related to a drug. These events can be preventable (e.g. wrong dose) or non preventable (e.g. rash due to an antibiotic). Non preventable ADEs are also called adverse drug reactions (ADRs). The World Health Organization (WHO) definition of ADRs excludes reactions associated with error, which are of greatest interest from the prevention perspective [11]. ADRs were not collected in this study.

*Seriousness of ADEs *was classified on categories according to WHO classification [23]: *Category A*: circumstances or events that have the capacity to cause error. *Category B*: an error occurred but the error did not reach the patient. *Category C*: an error occurred that reached the patient but did not cause patient harm. *Category D*: an error occurred that reached the patient and required monitoring to confirm that it resulted in no harm to the patient and/or required intervention to preclude harm. *Category E*: an error occurred that may have contributed to or resulted in temporary harm to the patient and required intervention. *Category F*: an error occurred that may have contributed to or resulted in temporary harm to the patient a required initial or prolonged hospitalization. *Category G*: an error occurred that may have contributed to or resulted in permanent patient harm. *Category H*: an error occurred that required intervention necessary to sustain life. *Category I*: an error occurred that may have contributed to or resulted in the patient's death.

### Statistical analysis

Data are summarized as mean ± standard deviation for variables with a normal distribution, median interquartile range (IQR) for variables with skewed distributions, and percentages for categorical variables. We reported rates of errors per 100 orders, 100 admissions and 1000 patient-day (Observation period of every patient included in the study, from the admission in the ICU to leaving it, transferring in other service or dying). Statistical analyses were carried out using SPSS version 13.0 (SPSS; Chicago, IL, USA).

## Results

### Study Population

During the study period, a total of 63 eligible study patients were admitted to the medical ICU. A total of 509 patient-days of admission, during which 4942 prescription episodes were written.

The mean age of the study patients was 49 ± 21 years and 37 (59%) were male. Mean APACHE II score was 11 ± 6. The median length of stay was 5 [4-10] days. Concerning the diagnosis categories, 21 (33%) were neurological disorders, 19 (30%) were respiratory disorders, and 8 (13%) were hepatic and renal disorders (Table 2)

**Table 2:** Epidemiological characteristics of the study patients

Characteristics	
**Age **(mean ± SD); years	49 ± 21
**Gender n(%)**	
Male	37 (59)
Female	26 (41)
**APACHE II **(mean ± SD)	11 ± 6
**Lengh of stay **(median [IQR]); days	5 [4-10]
**Diagnosis **n(%)	
Neurological disorders	21 (33)
Respiratory disorders	19 (30)
Hepatic and renal disorders	8 (13)
Infectious disorders	7 (11)
Intoxications	3 (5)
Others	5 (8)

APACHE II, Acute Physiology and Chronic Health Evaluation II score; IQR, interquartile range

### Identification of Medication-Related Events

A total of 4942 orders were reviewed, and prescription rates were 7844 per 100 admission and 9709 prescription per 1000 patient-days. We noted a total of 492 MEs. The incidence of MEs was 10 MEs per 100 orders, 780 MEs per 100 admissions, and 967 MEs per 1000 patient-days.

The frequency and rates of ADEs and potential ADEs were 113 potential ADEs, and 8 ADEs. The 113 potential ADEs occurred at a rate of 2.28 per 100 orders, 179.3 per 100 admissions, and 222 potential ADEs per 1000 patient-days. There were 8 ADEs, which corresponded to a rate of 0.16 ADEs per 100 prescription episodes, 12.6 ADEs per 100 admissions, and 15.7 ADEs per 1000 patient-days (Table 3).

**Table 3:** Incidence of Medication Errors reported to prescriptions and admissions

	Total	N/100 orders	N/100 admissions	N/1000 patient-days
**Orders**	4942	NA*	7844	9709
**Medication error**	492	10	780	967
**Adverse drug events**	8	0.16	12.6	15.7
**Potential adverse drug events**	113	2.28	179.3	222

*NA indicates data not applicable

### ME characteristics and seriousness of events

From 492 MEs, 60% were noted at the stage of transcription, and 35% at the stage of physician ordering. Concerning the type of MEs, 73% of MEs were wrong route of administration and 11% were wrong dose. Concerning the drug category of MEs, 33% were antibiotics and 31% were anticoagulants. From 113 potential ADEs, 60% were noted at the stage of transcription, and 33% at the stage of physician ordering. Concerning the type of potential ADEs, 73% were wrong route of administration and 11% were wrong dose. Concerning the drug category of potential ADEs, 33% were antibiotics and 31% were anticoagulants (Table 4).

**Table 4:** Characteristics of medication errors

Variables	Medication errors	Potential ADEs
	n = 492	n = 113
**Stage of errors, n(%)**		
Physician ordering	172 (35)	38 (33)
Transcribing	294 (60)	68 (60)
Administering	8 (2)	3 (3)
Dispensing	17 (3)	4 (4)
**Error type, n(%)**		
Omission	15 (3)	4 (3)
Dose	57 (11)	12 (11)
Frequency	38 (8)	9 (8)
Route of administration	357 (73)	82 (73)
Patient monitoring	7 (1)	2 (2)
Missing	18 (4)	4 (3)
**Drug category, n(%)**		
Anti-infective drugs	162 (33)	37 (33)
Anticoagulants	152 (31)	35 (31)
Corticoids	45 (9)	10 (9)
Analgesic and sedatives	20 (4)	5 (4)
Vasoactives drugs	25 (5)	4 (3)
Perfusion	6 (1)	2 (2)
Others	82 (17)	20 (18)

ADEs, Adverse Drug Events

Concerning the seriousness of events, Severity of ME was in 75% assessed on category A, and in 22% assessed on category B. Severity of potential ADEs was in 96% assessed on category B. From 8 ADEs, 5 ADEs required intervention necessary to sustain life (category H), and 2 ADEs were fatal (category I) (Table 5).

**Table 5:** Seriousness classification of medication errors (MEs), adverse drug events (ADEs), and potential ADEs

Classification	ME	ADEs	Potential ADEs
	(n = 492)	(n = 8)	(n = 113)
**Category A**	371	0	0
**Category B**	109	0	109
**Category C**	2	0	2
**Category D**	2	0	2
**Category E**	0	0	0
**Category F**	0	0	0
**Category G**	1	1	0
**Category H**	5	5	0
**Category I**	2	2	0

Seriousness of ADEs was classified on categories according to WHO classification [22]

## Discussion

In our study, medication errors were common in the inpatient medical ICU setting. Potential ADEs occurred more frequently than ADEs. The rate of medication errors was high. Errors occurred most commonly at the stage of drug ordering and transcribing. All types were concerned but those essentially increased were drug route and dosing errors. The drug classes associated most frequently with errors were anti infective and anticoagulants.

Comparing these results with those from a study by Bates et al [9] using similar methods in an adult patient population, our study had a higher rate of medication errors (10 errors/100 orders in our study vs. 5.3/100 orders). In a clinical review [24], the rate of medical errors among critically ill adults ranges from 1.2 to 947 errors per 1000 patient-days with a median of 106 errors per 1000 patient-days, the incidence of medication errors in our study seems then to be higher. However, a single centre study in a medical ICU [25] using an observation method of medication administration reported a higher rate with 1500 MEs per 1000 patient-days. In fact, a systematic review of ME incidence in different ICU types [26] have found a wide variation in reported rates of MEs. We believe thus, that much of this large variability was due to differences in the definitions used of the same type of event and also in methods used to detect events. Because most MEs do not result in harm, it is logical that MEs are more frequent than ADEs.

The incidence of ADEs and potential ADEs found in our study is consistent with what is expected based upon the literature. Several studies have focused on ME incidence and few data are available concerning ADE and portential ADE incidences [26]. To maximize the yield of events, we used a multifaceted approach to event detection involving chart review supplemented by other methods, based on that undertaken by Cullen et al [13]. In our study, the incidence of potential ADEs (222 per 1000 patient-days) was found to be about 20 times higher to that reported by Cullen et al [13] (13.5 potential ADEs per1000 patient-days). However, Rothschild et al [27] in a cardiac surgery ICU using also a multifaceted approach reported a rate of 23.8 potential ADEs per1000 patient-days which is lower to ours. Measuring ADE rates is also useful since this identifies actual situations in which patients are harmed and also allows for change for safer policies [26]. Concerning the incidence of ADEs, 15.7 ADEs per 1000 patient-days were noted in our study, Cullen et al [13] in a mixed ICU using a multifaceted approach reported a rate of 5.1 ADEs per 1000 patient-days. Rothschild et al [28] using the same method in a mixed ICU reported much higher rate (37.6 ADEs per 1000 patient-days). The exact rate of events is difficult to determine, but it has become widely accepted that the best estimate of the incidence of events requires comprehensive data collection using multiple strategies [26]. The incidence of events is greatly influenced by the definitions used, the method of detection and classification of events, and the study setting [19]. This makes comparison of reported rates between studies extremely difficult and, thus, any differences in rates should not be interpreted simply as reflecting differing levels of the quality of care between institutions, but more as reflecting differences between study methodologies.

Previous studies have not reported criteria for defining injury, and other studies have used multiple definitions for the same type of event [26]. The reason for this diversity of definitions is likely related to that fact that no standard definition is accepted by all the major organisations related to medication safety [26]. We used a multidisciplinary approach that examined all aspects of the medication system, from the physician's order through administration of the drug to the patient. Moreover, we encouraged voluntary reporting by emphasizing the role of systems problems in the origin of errors and by nurturing a blame-free environment.

Although 35% of errors in our study occurred in drug orders and 60% in drug transcribing many of these errors were detected and corrected prior to the order reaching the pharmacy. Cogent theories regarding the origin of errors (often categorized as human factor research) have been developed. Most investigators have focused on problems in health care delivery systems that predispose to error, rather than emphasizing the role of individuals [29,30]. Ongoing multidisciplinary analysis of incidents is important for developing further system improvements

There were some limitations of the present study, our study included only 63 admissions but 509 patient-days of admission were noted and 4942 prescriptions episodes were written which can be easily exploitable. The location was a general hospital; there may be some limitations to the generalizability of the results of the study to other types of healthcare facilities. The scope of the study was limited to MEs, ADEs and potential ADEs occurring in hospitalized patients, and did not include outpatients. It is however unlikely that any harmful events would have been missed as such events would have prolonged hospital stay or would have been identified through readmission of the patient. However, our study has the strength point to compare both the results of MEs and ADEs with the literature data.

Our review of the literature highlighted 2 important findings. First, medication error rates vary widely among clinical settings (both ICU and non-ICU settings), patient populations and studies. The reasons for this variation are likely multifactorial, but the reasons may include different patient populations (illness severity, number and type of prescriptions) clinical practice variation, lack of uniformity of definitions, the processes under investigation (e.g., prescription, transcription), methods of reporting and the culture of the different centers reporting their data [31]. The lack of standard definitions and reporting techniques make comparisons across organizations, regions or countries difficult. Second, although there are many potential risk factors for medication errors, the strongest evidence that critically ill patients are at increased risk of a medication error are increased severity of illness; failure to document the patient's usual medication list; prescription of cardiovascular, sedative, analgesic, anticoagulant or anti-infective medications; prescription of each additional medication; admission to a medical ICU compared with a surgical ICU [31].

Potential strategies to prevent medication errors in the ICU are focused on 7 prevention strategies: eliminating extended physician work schedules, computerizing physician order entry, implementing support systems for clinical decisions, computerizing intravenous devices, having pharmacists participate in the ICU, reconciling medications and standardizing medications [31].

The practical approach is to recognize that errors are a reality of medicine and that all health care providers have a responsibility to ensure patient safety and to use caution in promoting interventions. Improved medication safety may be accomplished by optimizing the safety of the medication process, eliminating situational risk factors and adopting strategies to intercept errors and mitigate their consequences.

## Conclusion

The medical use process itself is a complex system, providing many opportunities of ME occurring. Our incidence of ME was generally similar to that of others ICU studies which used similar methods of detection events. All types and categories were identified with high prevalence of transcribing error and wrong route, drugs incriminated was antibiotics and anticoagulants. These results can be used to improve quality of health care delivery; some authors recommended focusing on computerized approach like a first step to prevention in ICU in developing and developed countries. We believe that this improvement research is an expensive technology in our developed countries. We recommend standardization of therapeutic protocols and a systematic checking of prescriptions and transcriptions to reduce incidence of ME.

## Key messages

Medication errors in a medical ICU

## List of abbreviations

ADE: adverse drug event; ADR: adverse drug reaction; ICU: intensive care unit; IQR: interquartile range; ME: Medication error; WHO: World Health Organization.

## Competing interests

The authors declare that they have no competing interests.

## Authors' contributions

NJ drafted the manuscript, and participated in the acquisition of data. NM drafted the manuscript, participated in the acquisition of data and the study design. RO participated in the acquisition of data. KA helped to draft the manuscript, and participated in the acquisition of data. IK helped to draft the manuscript. JH participated in the coordination of the study. TD participated in the coordination of the study. AAZ participated in the design of the study, and performed the statistical analysis. RA conceived of the study, participated in the design of the study, performed the statistical analysis and interpretation of data, and gave the final approval of the manuscript. All authors read and approved the final manuscript
